# From Oncogenesis to Theranostics: The Transformative Role of PSMA in Prostate Cancer

**DOI:** 10.3390/cancers16173039

**Published:** 2024-08-31

**Authors:** Muhammad Y. Hameed, Maryam Gul, Abbas Chaudhry, Huma Muzaffar, Mubashir Sheikh, Winson Chee, Sondos Ayyash, Jenna Ayyash, Mohannad Al-Hindi, Humam Shahare, Ammar Chaudhry

**Affiliations:** 1 College of Medicine, University of Arkansas for Medical Sciences, Little Rock, AR 72223, USA; myhameed@uams.edu (M.Y.H.); wchee@uams.edu (W.C.); mal-hindi@uams.edu (M.A.-H.); hmshahare@uams.edu (H.S.); 2 Crescent Theranostics, Anaheim, CA 982902, USA; maryamgul@gmail.com (M.G.); abbas_chaudhry@berkeley.edu (A.C.); humamuzaffar9@gmail.com (H.M.); mubashir88@gmail.com (M.S.); 3 Department of Medical Oncology, University Health Network (UHN), Toronto, ON M5G 2C1, Canada; sondos.ayyash@uhn.ca; 4 Department of Biology, University of Toronto, Toronto, ON M5S 1A1, Canada; jenna.ayyash@mail.utoronto.ca; 5 Astrazeneca, Gaithersburg, MD 20878, USA

**Keywords:** Lutetium (177Lu) vipivotide tetraxetan, Pluvicto, prostate cancer, Actinium 225, Lutetium 177, theranostics, prostate-specific membrane antigen (PSMA)

## Abstract

**Simple Summary:**

Prostate cancer remainsa leading cause of cancer-related deaths in men worldwide. This review focuses on the transformative role of prostate-specific membrane antigen (PSMA) in the diagnosis and treatment of prostate cancer. PSMA is highly expressed in prostate cancer cells, making it a valuable target for both imaging and therapy. The integration of PSMA-targeted imaging with radioligand therapy, known as theranostics, has significantly improved the detection and management of advanced prostate cancer. Recent clinical trials, including the VISION trial, have demonstrated the effectiveness of PSMA-targeted therapies, such as 177Lu-PSMA-617. These advancements highlight the potential of theranostics in offering personalized and more effective treatment options for prostate cancer patients.

**Abstract:**

Prostate cancer, a leading cause of cancer-related mortality among men, is characterized by complex genetic and epigenetic alterations, dysregulation of oncogenic pathways, and a dynamic tumor microenvironment. Advances in molecular diagnostics and targeted therapies have significantly transformed the management of this disease. Prostate-specific membrane antigen (PSMA) has emerged as a critical biomarker, enhancing the precision of prostate cancer diagnosis and treatment. Theranostics, which integrates PSMA-targeted imaging with radioligand therapies, has shown remarkable efficacy in detecting and treating advanced prostate cancer. By leveraging the dual capabilities of PSMA-based diagnostics and therapeutic agents, theranostics offers a personalized approach that improves patient outcomes. This comprehensive review explores the latest developments in PSMA-targeted theranostics and their impact on the future of prostate cancer management, highlighting key clinical trials and emerging therapeutic strategies.

## 1. Oncogenesis 

Cancer is characterized by the abnormal uncontrolled proliferation of cells forming a tumor as a solid mass or infiltrating various tissues, including bone marrow. Tumors originating from hematopoietic cells is categorized as hematologic malignancy, while tumors originating from nonhematopoietic cells are termed solid tumors. Solid tumors account for most cancers and include carcinomas, sarcomas, and melanomas, whereas hematologic cancers include leukemia, lymphoma, and myelomas. 

Our understanding of the pathogenesis of cancer has evolved significantly over the years. Landmark papers include Boveri’s 1914 hypothesis of cancer’s cellular origin and Yamagiwa and Ichikawa’s 1918 demonstration that coal tar could induce cancer in animal models [1,2]. The identification of DNA’s structure by Watson and Crick in 1953 paved the way for understanding cancer at a molecular DNA level [3]. The Human Genome Project furthered our ability to study changes in genes and their involvement in cancer [4]. Fundamental characteristics that define cancer cells involves acquiring six biological capabilities: sustaining proliferative signaling, evading growth suppressors, resisting cell death, enabling replicative immortality, inducing vasculature, and activating invasion and metastasis [5,6]. In recent years, additional emerging hallmarks such as cellular metabolism dysregulation, inflammation, evading natural anti-tumor immune response, genomic instability, and continual acquisition of gain- or loss-of-function mutations add to the complexity of oncogenesis and the progression of cancer. A deeper understanding of oncogenesis is paving the way for precision medicine and is providing a framework for innovative targeted therapies such as immunotherapy, CAR-T cell therapy, and theranostics.

Genes contributing to oncogenesis are grouped into proto-oncogenes and tumor suppressor genes. Proto-oncogenes, such as *MET* and *MYC*, are involved in normal cell proliferation and differentiation, and acquired or genetic mutations in these genes leads to abnormal uncontrolled cell proliferation [7]. For example, *MYC* encodes the transcription factor *MYC*, which is the third most amplified gene in human cancers and is overexpressed in several cancer subtypes, including prostate cancer [8,9]. Meanwhile, tumor suppressor genes regulate the cell cycle and control cellular proliferation. The *TP53* gene regulates cell apoptosis and cycle progression, and mutations or the loss of TP53 results in hematologic (lymphomas, leukemias) and solid cancers (prostate, breast, lung) [10]. *BRCA1* and *BRCA2* are two tumor suppressor genes that are involved in DNA repair, and alterations in BRCA1/2 increase the likelihood of epithelial malignancies, including prostate cancer [11]. Mutations in proto-oncogenes and tumor suppressor genes can be inherited or acquired from environmental factors, such as exposure to carcinogens like aflatoxin, which causes mutations in the P53 gene, leading to liver cancer [12]. Deregulation of epigenetic changes, such as DNA methylation and histone modifications, can also contribute to cancer development [13]. Additionally, exogenous sources, like radiation and smoking, and endogenous sources, such as depurination, deamination, and oxygen radicals, can cause DNA damage and lead to cancer [14]. Considering the diverse pathways leading to oncogenesis and the intricate nature of solid tumors, this review will concentrate specifically on prostate cancer. It will delve into the latest advancements in precision medicine within prostate cancer, including novel diagnostic techniques (PSMA-PET), and explore the role of theranostics in the treatment of prostate cancer.

## 2. Introduction to Prostate Cancer

Prostate cancer is the most commonly diagnosed malignancy amongst men and is the fifth leading cause of cancer-related mortality in men [15]. Based on GLOBOCAN estimates, 1,414,259 new cases of prostate cancer were reported worldwide in 2020 [16]. Incidence and mortality rates vary considerably across different geographical locations [17]. This variability may be attributed to differences in the implementation of detection strategies reliant on prostate-specific antigens (PSAs), which has become the gold standard for prostate cancer screening. Prostate cancer screening has led to the improved diagnosis of early-stage prostate cancer and has consequently improved survival [18]. 

Most patients diagnosed with prostate cancer present with localized, prostate-limited disease, which is highly curable. However, metastasis, whether presenting at diagnosis or after treatment, significantly reduces survival, contributing to more than 375,000 prostate cancer-related deaths globally in 2020 [19]. Prostate cancer usually first metastasizes to lymph nodes surrounding the prostate, and then may spread to the bone and distant lymph nodes [20]. The liver, however, is the most fatal site of castration-resistant prostate cancer, with a median survival of 10 to 14 months [21].

The molecular biology underlying prostate cancer oncogenesis is complex, involving a variety of genetic and epigenetic alterations. Central to this process is the dysregulation of androgen receptor (AR) signaling, which is crucial for the growth and survival of prostate cancer cells. The AR, upon binding with androgens like testosterone and dihydrotestosterone (DHT), activates a transcriptional cascade that promotes tumor progression [22]. Genetic mutations also play a major role in this disease, with alterations in key tumor suppressor genes such as PTEN and TP53 being common [23]. PTEN loss leads to the unchecked activation of the PI3K/AKT pathway, promoting cellular survival and proliferation, while TP53 mutations result in genomic instability and resistance to apoptosis [24,25]. Furthermore, oncogenic drivers, like the MYC gene and TMPRSS2-ERG gene’s fusions, contribute to the aggressive behavior of prostate cancer cells [26]. Epigenetic changes, including DNA methylation and histone modifications, which regulate gene expression, add another layer of complexity to the disease’s molecular landscape [27]. The study of these molecular mechanisms is vital for developing targeted therapies and improving patient outcomes in prostate cancer.

The classification of a prostate cancer diagnosis is determined based on a combined analysis of tumor-node metastasis staging, PSA, and the Gleason score, which stratifies prostate cancers from low-grade to high-grade disease. According to this score, Grade 1 indicates well-differentiated low-grade dysplastic tissue, whereas Grade 5 signifies the most abnormal dysplastic tissue [28].

## 3. PSMA and Prostate Cancer Diagnosis

Historically, prostate cancer screening has been performed using a combination of clinical symptoms, digital rectal examinations, and prostate-specific antigen (PSA) levels in the blood. However, elevated PSA levels can be found in many prostate diseases such as prostate cancer, prostatitis, and benign prostate hyperplasia. Thus, elevated PSA levels between 4 and 10 ng/mL are only associated with prostate cancer in approximately 25% of cases [29,30]. Additionally, 15% of patients with prostate cancer have normal PSA levels below 4 ng/mL [24,25]. Therefore, imaging and biopsy are of vital importance to prostate cancer diagnosis.

Transrectal ultrasound (TRUS) was the first imaging technique used for localizing prostate cancer and assisting in biopsy. While TRUS-guided biopsies were cost-effective, they had shortcomings including invasiveness, risk of infection, and the high potential for missed cancers [31]. The use of magnetic resonance imaging (MRI) for tumor staging and biopsy improved the detection rate over TRUS; however, MRI-guided biopsies still only detected approximately 40–60% of prostate cancers [32]. The adoption of multiparametric MRI and the Prostate Imaging Reporting and Data System (PI-RADS) guidelines stratified prostate cancer lesions to assist in detection and clinical decision-making [33]. Biopsies are generally indicated in PI-RADS 3 or greater lesions, and the use of multiparametric MRI helped to avoid unnecessary biopsies and decreased low-grade cancers or benign tissues obtained from the biopsies that have been performed [28]. However, these techniques have not greatly improved detection rates using MRI alone.

The development of PET and radioisotope-based imaging combined with CT or MRI greatly increased the sensitivity and specificity for prostate cancer diagnosis and surveillance. The most widely used PET radiotracer is F-18 fluorodeoxyglucose (FDG), which was first synthesized and used for imaging in the 1970s. FDG is preferably taken up by cells with high glucose consumption, and is widely used in the detection of many types of malignancies. However, prostate cancer is typically slow growing and has lower glucose transporter levels than other types of malignancies [25]. Thus, low tumor-to-background ratios result in a decreased detection rate of prostate cancer using FDG PET [25]. 11C-Choline was first used as a PET tracer for prostate cancer in 1998 and demonstrated a better detection rate than MRIs at the time [34]. However, prostate cancer recurrence was only detected in 55% of patients, and the detection rate of prostate cancer with low PSA values (less than 1.16 ng/mL) was only 27% [35]. Although 11C-choline was FDA approved for recurrent prostate cancer PET scans in 2013, it is no longer widely used compared to targeted radiotracers for prostate cancer due to its inferior detection rates when PSA is low [36]. In 2016, 18F-FACBC (F18-fluciclovine) was FDA approved for recurrent prostate cancer. This molecule is an analog of leucine and accumulates in tumor cells that upregulate amino acid transporters. However, while the detection rate of 18F-FACBC was improved compared to 11C-choline in patients with recurrent prostate cancer, it also had lower detection rates for patients with low PSA [37]. Additionally, poor detection of metastases smaller than 5mm limited the use of F18-FACBC in prostate cancer staging [38]. Given the poor ability of traditional methods and general radiotracer ligands to detect prostate cancer, research shifted to target proteins specific to prostate cells. 

PSMA is a transmembrane glycoprotein encoded by the FOLH1 (folate hydrolase 1) gene, located on chromosome 11p11.12 [39,40]. It is not only expressed in malignant prostate tissue, but also benign prostate cells and other tissues, including the salivary glands and renal cortex [41]. PSMA is expressed at much higher rates in prostate cancer cells, with studies showing up to a 1000-fold increase in expression [42,43]. Higher expression of PSMA in prostate cancer cells has been associated with poorer disease outcomes and is also linked to hormone-therapy-resistant cancer [44,45]. 

The role of PSMA in prostate cancer oncogenesis and proliferation is complex and not fully understood [46]. PSMA has enzymatic activity, including glutamate carboxypeptidase and folate hydrolase [47]. PSMA’s ability to hydrolyze N-acetylaspartylglutamate into N-acetylaspartate and glutamate is of particular importance. Glutamate as a signaling molecule that has been shown to activate the PI3K/AKT pathway in prostate cancer cells, maintaining telomere stability in malignant cells [46,48]. Knockdown of PSMA has also been shown to cause significant metabolic disruptions and alterations in the biosynthesis pathways of arginine and proline, which in turn affect the proliferation and invasion capabilities of prostate cancer cells and transcriptional changes that include the downregulation of the androgen receptor (AR) signaling pathway and the upregulation of oncogenes such as c-Fos and FosB [47]. In addition, PSMA also plays a role in the degradation of the extracellular matrix (ECM) by regulating matrix metalloproteinases, such as MMP7 and MMP9, increasing ECM breakdown and promoting tumor invasion [47]. PSMA overexpression in prostate cancer cells, which are particularly sensitive to folate-depleted conditions, enhances their survival in folate-depleted conditions by increasing the uptake of folic acid and converting polyglutamated folates into more easily absorbed monoglutamated forms [49,50,51]. 

PSMA-targeting ligands used in PET have significantly enhanced the detection and management of prostate cancer. While other prostate-cancer-targeting tracers have been studied, such as prostate stem cell antigens, gastrin-releasing peptide receptors, and urokinase plasminogen activators, among many others, PSMA has had the most promising history. The first radiotracer, In111-capromab pendetide, was FDA approved for prostate cancer imaging in 1996. It utilized the 7E11-C5 antibody and targeted an intracellular domain of PSMA in dead or dying cells, which led to poor sensitivity [25,35]. Antibodies such as J591 were subsequently developed to target the extracellular domain in viable cells []. However, the images obtained were of poor resolution, and patients suffered from side effects such as marrow toxicity [34].

Finally, researchers worked to synthesize smaller molecules (low molecular weight ligands) targeting PSMA. Ga68-Ga-PSMA-11 was FDA approved in 2020 for patients suspected of prostate cancer metastases or recurrence. It demonstrated similar detection rates as C11-choline and F18-FACBC when PSA was less than 2.2 ng/mL, but significantly improved detection rates when PSA was greater than 2.2 ng/mL [52]. F18-DCFPyL was FDA approved in 2021 after being derived from F18-DCFBC. Fluorine-18 has a longer half-life and wider availability than gallium-68 and has been shown to be safe and effective in prostate cancer diagnosis [25,35]. Additionally, when compared to F18-NaF, which has been historically used to detect bony lesions in prostate cancer, sensitivities were similar, but F18-DCFPyL provided much more information on the soft tissues (Rowe). Finally, in 2023, the FDA approved 18F-Flotufolastat (18F-rhPSMA-7.3) for use in PET for prostate cancer and its recurrence following the results of the LIGHTHOUSE (NCT04186819) and SPOTLIGHT (NCT04186845) studies [53,54]. These studies indicate that 18F-rhPSMA-7.3 has high detection rates of prostate cancer from a wide variety of PSA levels and is well tolerated by patients [36,37]. Additionally, the radiotracer is reported to have lower urinary excretion than traditionally used PSMA-PET agents, and the studies suggest an improved distinction between primary tumor and background bladder activity to aid in local tumor and lymph node identification [36,37].

## 4. PSMA PET Imaging

**Figure 1:**
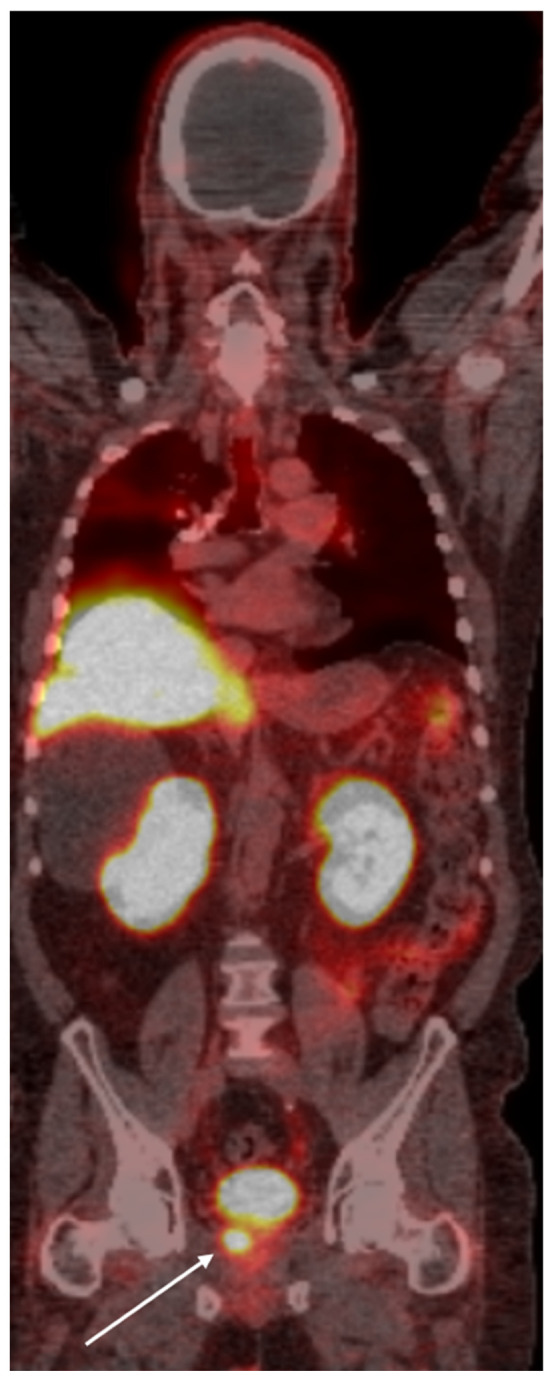
PSMA PET scan of a 66-year old demonstrates radiotracer uptake lesion in the right prostate bed (arrow), consistent with primary prostate carcinoma.

**Figure 2:**
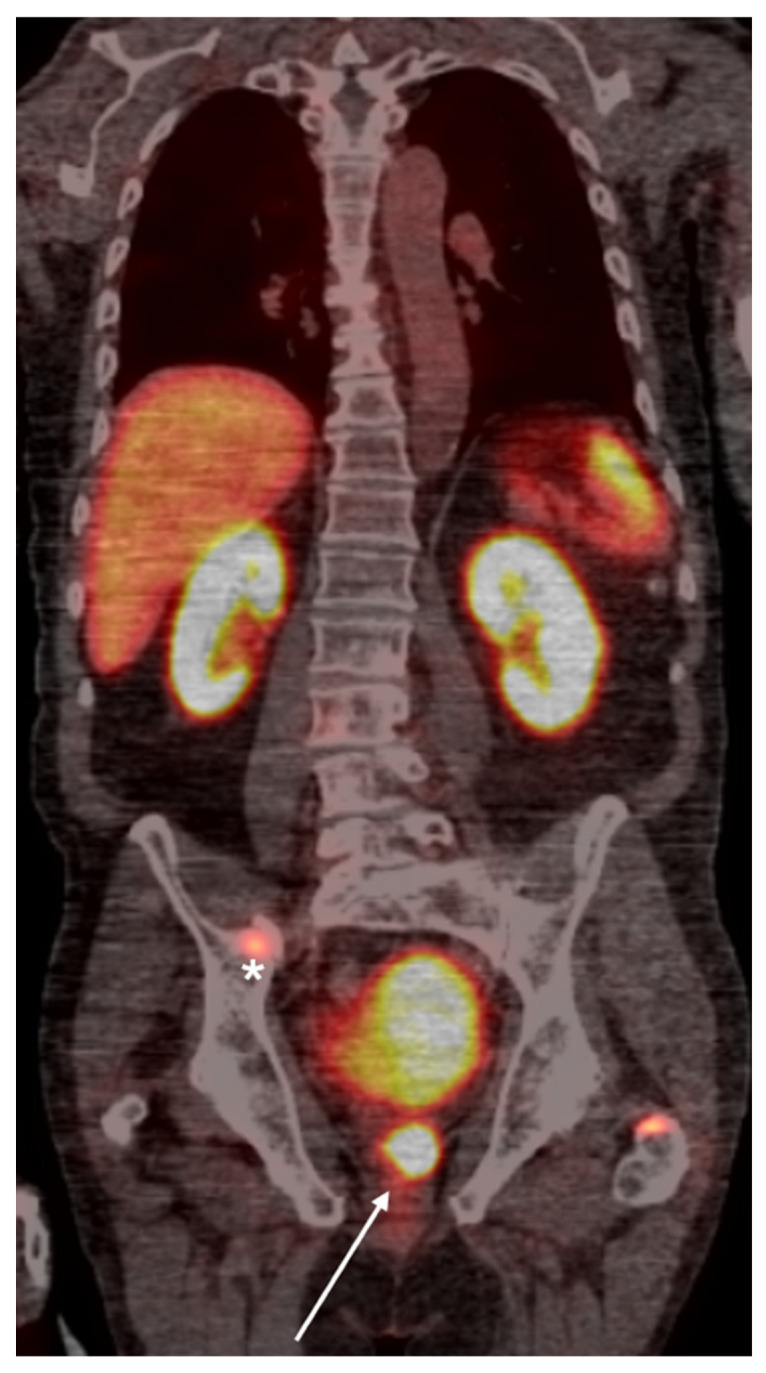
Whole-body PSMA PET scan demonstrating a primary lesion in the left prostate gland (arrow), with evidence of osseous metastasis in the pelvic bones (asterisk).

Recent advancements in PSMA-targeted imaging have transformed prostate cancer diagnosis and staging. Agents like 68Ga-PSMA-11 and 18F-DCFPyL, binding specifically to Prostate-Specific Membrane Antigen (PSMA), have revolutionized the field [55]. Compared to standard imaging methods such as bone scans, CT, and MRI, PSMA-targeted imaging, like PSMA PET/CT, offers superior sensitivity and specificity in identifying prostate cancer, especially in the initial staging of prostate cancer (Figure 1 and Figure 2). This comprehensive approach aids in assessing visceral, bone, lymph node, and primary tumor metastases in a single examination, impacting clinical care in approximately 25% of patients [41]. Additionally, combining PSMA PET with multiparametric MRI enhances sensitivity in detecting seminal vesicle invasion and extra-prostatic extension [41]. 

PSMA-targeted imaging significantly outperforms traditional techniques like bone scintigraphy and CT scans, particularly in detecting early-stage disease, leading to improved staging accuracy and more effective treatment planning and management [56,57]. Moreover, recent research highlights significant therapeutic advantages of PSMA-targeted imaging, potentially transforming patient care through enhanced disease detection, crucial for patients experiencing biochemical recurrence and those making treatment decisions [43].

Research consistently demonstrates the superior performance of PSMA-targeted imaging agents compared to other molecular tracers, such as fluoride, fluciclovine, and choline, particularly in patients with biochemical recurrence of prostate cancer. Notably, 68Ga-PSMA PET shows higher detection rates than 18F-Choline PET, with a greater clinical treatment impact [58,59]. Moreover, PSMA agents offer a better contrast for metastases to the skeleton and lymph nodes, enhancing staging accuracy and treatment planning, and are more accessible and economical due to the availability of 68Ga from a radionuclide generator.

PSMA-targeted imaging, exemplified by 68Ga-PSMA HBED-CC, provides improved accuracy in detection and diagnosis by precisely targeting PSMA, which is highly expressed in prostate cancer cells [60]. These agents offer higher selectivity compared to multiparametric MRI (mpMRI) and better distinguish between malignant and benign tissues. Combined with MRI, PSMA-targeted agents enhance both specificity and sensitivity, making them more effective tools for comprehensive and accurate prostate cancer diagnosis and treatment [46]. 

Furthermore, PSMA-targeted imaging demonstrates higher sensitivity and specificity than conventional choline-based tracers like 11C-choline and 18F-choline in identifying recurrent prostate cancer, particularly at low PSA levels [61,62]. This improved performance is crucial for the early detection and management of recurrent prostate cancer, guiding patient-specific treatment strategies effectively. Therefore, PSMA-targeted imaging agents are preferred over other nuclear medicine compounds for managing prostate cancer recurrence post-prostatectomy with low PSA levels [63]. 

Preclinical studies have shown that androgens downregulate the FOLH1 gene, which expresses PSMA, and androgen deprivation therapy, which, on the other hand, increases PSMA expression by upregulating the FOLH1 gene [64,65]. Hope et al. in 2017 was the first clinical report to show an increase in PSMA uptake on 68GA-PSMA-11 after initiation of ADT, raising the possibility of increasing the sensitivity of detecting metastases after ADT [65 A clinical trial (NCT03313726) showed that an increase in PSMA uptake post-ADT was heterogenous and had a minor effect on the performance of 68Ga-PSMA-11 PET in increasing detection, having a significant clinical impact [66]. A prospective study of hormone-naïve prostate cancer patients who received 3 months of ADT showed a significant reduction in PSMA PET/CT indices [67]. The consensus statement on the PSMA PET/CT response assessment advises that due to potential flares of ADT, PSMA PET/CT should not be performed earlier than 3 months after the start of ADT, and the statement also acknowledges that the relationship between PSMA expression and ADT is complex and requires more study [68,69]. 

PSMA PET imaging, while highly effective, has limitations, including the potential for false-positive results due to non-specific PSMA uptake. PSMA uptake in bone is common in advanced disease associated with increased vascularity and bone remodeling and also with benign conditions such as fibrous dysplasia, fractures, and Paget’s disease [70,71,72]. Additionally, lymph nodes, which are the second most common site of prostate cancer metastasis after bone, often show physiologic uptake in mediastinal lymph nodes, but the radiotracer uptake has been shown to be much lower than malignant nodes [73]. 

## 5. Theranostics

Theranostics is an innovative field in precision medicine that integrates diagnostic techniques with therapeutic interventions, specifically using radiopharmaceuticals for targeted molecular imaging and treatment. The term itself is derived from a combination of two words: ‘therapeutic’ and ‘diagnostic.’ As a recently developed field of nuclear medicine, it allows for precise treatment using either a single radionuclide for both imaging and therapy, or separate radionuclides for each process. A vector is used to bind to a target on a cell and the vector is attached to a radionucleotide via a linker molecule. The location of the cells can be visualized with imaging, enabling the confirmation and selection of patients for whom target radionuclide therapy may be suitable, as well as determining the required dosing. Although the term ‘theranostics’ was first noted in the medical literature in the early 2000s, the concept itself is not new. An example of this is radioactive iodine, which has been used since the 1940s to treat thyroid disease [74,75]. Over the decades, this approach has evolved into an established treatment for both benign and malignant thyroid conditions [76]. More recent advancements were made in the 1990s for treating neuroendocrine tumors with In-111 and Ga-68 somatostatin analogs coupled with Y-90 and Lu-177 [77]. The NETTER trial in 2017 led to the FDA approval of Lu-177 DOTATAE for neuroendocrine tumors [78].

**Figure 3:**
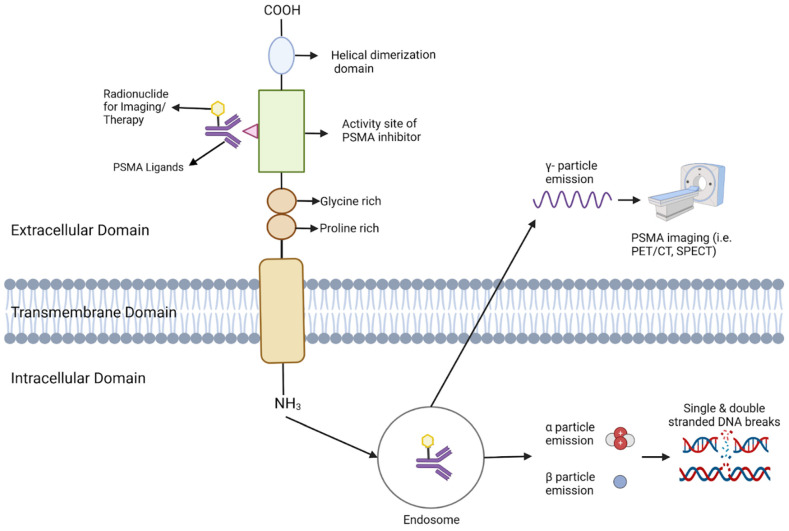
This Figure depicts the configuration and operation of PSMA (Prostate-Specific Membrane Antigen) in targeted imaging and treatment. The PSMA receptor extends across the cellular membrane, comprising an external, transmembrane, and internal domain. Ligands bind to the external domain, initiating the receptor-ligand complex’s internalization into endosomes. Within the endosome, the radionuclide remains bound and emits various particles depending on its nature. γ-particles are emitted for imaging purposes, facilitating visualization via PET/CT or SPECT. For therapeutic use, α and β particles are emitted, inducing DNA damage that selectively destroys cancer cells.

After binding to the extracellular site, PSMA ligands are internalized and subsequently release gamma, beta, or alpha particles (Figure 3) [79]. Both beta and alpha particles are primarily utilized for therapeutic purposes. Beta particles, such as those emitted by 177 Lu, have lower ionizing energy and longer tissue penetration compared to alpha particles. Beta particles from 177 Lu exhibit moderate ionizing energy ranging from 0.05 to 0.5 MeV, whereas alpha particles from 223 Ra 223 Ra, the most tested alpha emitter for prostate cancer, has a mean energy of 6.95 MeV [80]. The tissue penetration of these particles also differs significantly: 177 Lu has a mean range of 0.28 mm in soft tissue, while 223 Ra has a much shorter range of 0.054 mm [56]. These distinct properties make beta and alpha particles suitable for different therapeutic applications. Beta particles, due to their longer travel distances, can cover a larger area of tissue, but may also pose a higher risk of damaging surrounding healthy cells. This can be useful in larger tumor masses, where the crossfire effect may prove to be beneficial [81]. The higher cytotoxicity of alpha particles and increased precision of radiation delivery theoretically makes it suitable for smaller tumors [82].

The history of PSMA theranostics for prostate cancer began with Y-90-capromab pendetide, the first theranostic agent developed for this disease. Capromab pendetide (7E11-C5.3) is a monoclonal antibody that targets the intracellular domain of PSMA and was initially radiolabeled with indium-111 for imaging purposes [83]. It received FDA approval in 1996 for prostate cancer imaging. However, since it targets the internal domain of PSMA, it was ineffective against viable cancer cells, making it the only molecular radiotherapy for prostate cancer with this limitation. Phase 1 and 2 clinical trials revealed no therapeutic benefit and reported significant bone marrow toxicity [84,85].

## 6. J591

J591, the next antibody tested, targeted the extracellular domain of PSMA. Targeting the extracellular domain allows for the treatment to target healthy living cells, whereas targeting the intracellular domain is effective only when the cells are dying or dead. Early trials with 111In/Y90 showed promising results with good tumor response and biologic activity [86]. Future trials investigated 177Lu instead of Y90, as it theoretically offers a longer half-life and shorter beta emission range, which is beneficial in treating small volume prostate cancer metastases [87].

A phase 1 trial of Lu-177-J591 with 35 patients showed biologic activity, with most patients experiencing PSA decline or stabilization [63]. The maximum tolerated dose was 70 mCi/m^2^, limited by myelosuppression, and multiple doses of 30 mCi/m^2^ were well-tolerated. In a phase 2 trial with 47 patients, those receiving 70 mCi/m^2^ had higher rates of >30% PSA decline (46.9% vs. 13.3%) and longer overall survival (46.9% vs. 13.3%) than the 65 mCi/m^2^ group, but experienced increased grade 4 hematologic toxicity [88]. These trials demonstrated a dose-dependent relationship between 177Lu-J591 dose and treatment response and hematologic toxicity. A phase 1/2 trial with 49 patients investigated fractionated doses of Lu-177-J591 and showed that fractionated dosing allows for a higher overall cumulative dose with improved treatment response at higher doses [89]. These findings highlight the efficacy of 177Lu-591 in treating prostate cancer, revealing a dose-dependent relationship between treatment efficacy and toxicity, suggesting that dose fractionation may be a viable strategy.

Monoclonal antibodies like J591 have unique benefits, including lower salivary gland toxicity and reduced renal toxicity, as their larger size prevents renal and salivary gland penetration and common side effects like xerostomia, which is prevalent with small molecule ligands like PSMA-617 [90]. TLX591, a structurally modified version of J591, is now being tested in multiple trials (Table 1). Initial results from the phase 1 trial of 177Lu-TLX951 with SoC with 28 patients showed 18% of patients with a 50% PSA reduction and 27% with a 30% reduction (NCT0786847) [91]. Grade 4 hematologic toxicity was limited with thrombocytopenia (25%) and neutropenia (4%). Phase 2 and 3 trials with 177Lu-TLX591 are currently ongoing (NCT04876651, NCT05146973).

## 7. 177. LuPSMA-617

The VISION trial (NCT03511664) led the FDA-approved 177Lu-PSMA-617 for prostate cancer, making it the only approved radioligand for prostate cancer to date. The VISION trial was a phase 3 trial with 831 patients with PSMA-positive mCRPC [92]. In total, 551 patients were randomized to the group receiving 177Lu-PSMA-617 plus standard of care (SoC), versus 280 who received SoC alone. Eligibility criteria included patients who had experienced disease progression after treatment with androgen receptor pathway inhibitor (ARPI) with taxane, had an Eastern Cooperative Oncology Group performance status score between 0 and 2, expected life span of at least six months, and adequate bone marrow function. SoC excluded patients who had received cytotoxic chemotherapy, immunotherapy, systemic radioisotopes, and investigational drugs. Patients in the 177Lu-PSMA-617 group received infusions of 7.4 GBq (200 mCi) once every 4 weeks, with the option of an extra 2 cycles. Results showed an increased progression-free survival of 8.7 months in the experimental group versus 3.4 months in the control group. Overall survival was higher for the experimental group at 15.3 months versus 11.3 months. Incidence of grade 3 or higher adverse events was higher with 177Lu-PSMA-617 (52.7% versus 38%), with fatigue, nausea, and dry mouth being the most common adverse effects, but quality of life measurements remained favorable.

This approval for Lu177-PSMA-617 applies specifically to patients with mCRPC who have undergone at least one line of ARPI therapy and at least one taxane-based chemotherapy. Since its approval in North America and Europe in 2022, 177Lu-PSMA-617 has been adopted into prostate cancer management guidelines (Table 2) [93,94]. Around 34,000 patients are estimated to require this treatment annually in the United States alone [95]. The approval for Lu177-PSMA-617 is a second- or third-line agent, but trials are currently investigating its use in combination with other therapies earlier during the disease or even as first-line agents.

The UpFrontPSMA trial (NCT04343885), a phase II study in Australia, includes 140 newly diagnosed metastatic prostate cancer patients due to receive either sequential 177Lu-PSMA-617 (7.5 GBq every 6 weeks for two cycles) followed by docetaxel, or docetaxel alone in the control arm, with both groups receiving continuous ADT [96]. The LuTectomy trial (NCT04430192) is a phase I/II study evaluating 177Lu-PSMA-617 in patients with high-risk localized or locoregional advanced prostate cancer before radical prostatectomy and pelvic lymph node dissection, and demonstrated that two cycles of 177Lu-PSMA-617 prior to surgery were tolerated with minimal adverse effects [97]. The LuPSMA trial (ACTRN12615000912583) was phase II study with 30 patients with metastatic CRPC treated with up to four cycles of Lu177-PSMA-617 at 7.5 GBq at six week intervals [98]. In total, 57% of participants had a PSA reduction of 50% or more. The most common adverse effect was grade 1 dry mouth (87%), and grade 3/4 thrombocytopenia was reported in 13% of patients. 

Other major trials that have investigated 177Lu-PSMA-617 as a second-line treatment option include the TheraP trial and PSMAfore trial. The TheraP trial (NCT03392428) was a phase II clinical study involving 200 patients with mCRPC who had previously been treated with docetaxel [99]. Patients were randomized to receive either 177Lu-PSMA-617 or cabazitaxel. There was a higher PSA response rate in the 177Lu-PSMA-617 group, where 66% of patients saw over a 50% reduction in PSA levels, compared to 37% in the cabazitaxel group. The study also indicated superior progression-free survival with 177Lu-PSMA-617, despite similar overall survival rates between the two groups. The 177Lu-PSMA-617 group experienced fewer severe adverse events compared to the cabazitaxel group. The PSMAfore trial (NCT04689828) is a phase III trial involving 468 taxane-naïve mCRPC patients who have failed ARPI treatment [100]. Participants are randomized to receive either 177Lu-PSMA-617 or a change of ARPI therapy to abiraterone or enzalutamide. In preliminary data presented at ESMO 2023, the Lu177-PSMA-617 group had prolonged rPFS. In total, 57.6% of patients treated with Lu-PSMA-617 experienced a reduction in PSA levels by 50% or more, compared to 20.4% in the control group. The experimental arm experienced lower grade 3 or higher events at 33.9% versus 43.1%. Serious adverse events were also lower at 20.3% in the experimental arm versus 28.05% in the control. Treatment discontinuation due to adverse events was similar, 5.7% for Lu-PSMA-617 and 5.2% for ARPI. These studies demonstrate that Lu177-PSMA-617 not only yields improved outcomes when used as a second- and third-line treatment, but also exhibits lower toxicity compared to other options. This finding is significant, as ongoing trials exploring the use of 177Lu-PSMA-617 in combination therapies or as a first-line treatment show great promise for advancing prostate cancer care (Table 3).

## 8. 177. Lu-PSMA-I&T

Two major phase III trials are underway investigating 177Lu-PSMA-I&T. The SPLASH trial (NCT04647526) involves 412 mCRPC patients after receiving at least one APRI therapy without any chemotherapy. Patients were to receive either 177Lu-PSMA-I&T or either abiraterone or enzalutamide. Topline results show a median rPFS of 9.5 months for the 177Lu-PSMA-I&T arm and 6 months in the control arm [101]. A significant reduction of 29% in the risk of radiographic progression or death was observed. 177Lu-PSMA-I&T demonstrated a favorable safety profile with lower grade 3 adverse effects (30.1% vs. 36.9%). Additional data are expected in 2024, as well as a potential new drug application. The second phase III trial, the ECLIPSE trial (NCT05204927), includes 400 mCRPC patients randomized to either 177Lu-PSMA-I&T or standard care with abiraterone or enzalutamide. The primary endpoint for ECLIPSE is radiographic PFS, with secondary endpoints including OS, time to first symptomatic skeletal event, and health-related quality of life (HRQoL).

## 9. Ra-223

Ra-223 is currently the only FDA-approved alpha emitter used for prostate cancer. It was approved by the FDA and EMA in 2013 as result of the ALSMYPCA trial [102,103]. Ra-223 selectively targets hydroxyapatite, and thus it is beneficial in treating osteoblastic lesions in prostate cancer [104]. Earlier phase I and II trials showed efficacy in skeletal-related events and pain [105,106,107,108]. The ALSMYPCA trial was a phase III trial which included 921 mCRPC patients who had not received docetaxel and had symptomatic bone metastases without visceral lesions [79]. All patients received SoC and were randomized to either Ra-223 or placebo. Ra-223 provided an overall survival benefit and increased time to first symptomatic skeletal event, leading to FDA approval for mCRPC. Interim results of REASSURE, a prospective observational study in patients with mCRPC receiving RA-223 with median follow-up of 1 year, shows a comparable safety profile to that seen in ALSYMPCA [109].

Additional trials have looked at the use of Ra-233 in combination with abiraterone and enzalutamide. ERA-223 was a phase III trial with 806 patients randomized to receive Ra-223 or placebo [110]. All patients received abiraterone and either prednisone or prednisolone. The addition of Ra-223 did not improve symptomatic skeletal event-free survival, and an increase in bone fractures was seen in the Ra-223 group. This led to the EUA recommending a contraindication on the use of Ra-223 with abiraterone and prednisone or prednisolone [111]. PEACEIII is an ongoing phase III trial investigating the use of Ra-223 with enzalutamide. Safety and efficacy results do not show an increase in adverse effects and fractures [112]. This safety profile is promising, but the survival benefits are yet to be determined. 

## 10. Ac-225

Ac-225 is another alpha-emitting radionucleotide being investigated for use in prostate cancer. The first report of use of 255Ac-PSMA-617 was by Kratochwil et al. in 2016 in two patients both experiencing PSA decline and complete responses to imaging [113]. Notably, one of the patients had already failed 177Lu-PSMA-617 treatment. Xerostomia was the only clinically reported side effect, which has also shown to be prevalent in other studies [114,115]. A single course of low activity of Ac225-PSMA-617 combined with full activity 177Lu-PSMA-617 has been shown to have tolerable levels of xerostomia in 20 patients who had an insufficient response to 177Lu-PSMA-617 [116]. In a retrospective review of 106 patients who received Ac225-PSMA-617 for mCRPC with skeletal metastases, 80.2% of patients had a PSA response and a very low incidence of grade 3 and 4 hematologic toxicity [117]. In a retrospective study of 53 patients who received Ac225-PSMA-617 with mCRPC after ADT, 91% of patients had a >50% PSA decrease, and a >50% decrease in PSA was predictive of PFS and OS. Grade 1/2 xerostomia was seen in 81% of patients, but no severe hematologic toxicity was observed [118]. In a study of patients with de novo mHSPC, Ac225-PSMA-617 had favorable outcomes, with 86% of patients experiencing a >50% PSA decline [119]. The ongoing ACTION trial is a phase I trial of patients receiving 225Ac-PSMA-617 with or without prior 177-Lu-PSMA-617 treatment. Ac-225 holds potential as an alpha emitter therapy for mCRPC and mHSPC, and also in patients who may have had inadequate response to 177-Lu-617.

## 11. Conclusions

Oncogenesis is a complex process that varies across different types of tumors. Recent advancements have identified cell surface markers specific to certain tumors, leading to the development of precision medicines. Theranostics is a growing area of research that uses radioconjugate technology to target tumor-specific markers for both diagnostic and therapeutic purposes. One example of this is the use of prostate-specific membrane antigen (PSMA)-targeted imaging and theranostics, which has significantly improved the diagnosis and management of prostate cancer. PSMA, which is highly expressed in prostate cancer cells, is used as a biomarker for both diagnostic and therapeutic applications. PSMA-targeted imaging techniques, such as PSMA PET/CT scans with radiotracers like 68Ga-PSMA-11 and 18F-DCFPyL, provide increased sensitivity and specificity in detecting prostate cancer, aiding in tumor localization, staging, and treatment planning. Clinical trials, like the VISION trial, have validated the effectiveness of radioligand therapy with agents like 177Lu-PSMA-617 in extending progression-free and overall survival in patients with metastatic castration-resistant prostate cancer (mCRPC). Several novel ongoing theranostics studies are ongoing and are geared to deliver on the promise of precision medicine.

## Figures and Tables

**Table 1:** Summary of clinical trials assessing 177Lu-labeled therapeutics in prostate cancer.

Clinical Trial Identifier/Authors	Study Objective	Phase	Status
Bander et al. [71]	177Lu-J591 in androgen-independent prostate cancer	1	Complete
NCT00195039	177Lu-J591 in mCRPC	2	Complete
NCT00538668	177Lu-J591 in mCRPC	1/2	Complete
NCCT04786847 (ProstACT SELECT)	177Lu-TLX591 with SoC in mCRPC	1	Complete
NCT05146973 (ProstACT Target)	177Lu-TLX591 with external beam radiation therapy in biochemically recurrent, oligometastatic PSMA prostate cancer	2	Active, not recruiting
NCT04876651 (PROSTACT)	177Lu-TLX591 with Soc versus Soc alone in mCRPC	3	Not yet recruiting

**Table 2:** Criteria for assessing prostate cancer treatment responses.

Criteria	Full Name	Details
RECIST	Response Evaluation Criteria in Solid Tumors	Evaluates tumors’ reactions to treatments by monitoring changes in their size through anatomical imaging techniques. It primarily focuses on measurable tumor lesions.
PERCIST	PET Response Criteria in Solid Tumors	Assesses metabolic response to treatments using PET scans by monitoring changes in standardized uptake values (SUV) of tumors, emphasizing metabolic changes over size alterations.
PCWG3	Prostate Cancer Working Group 3	Provides guidelines tailored to prostate cancer for assessing treatment response and disease progression by considering PSA levels, imaging results, and clinical status.
PPP	PSMA PET Progression	Focuses on disease progression in PSMA PET, monitoring biochemical or clinical progression along with lesion counts observed through PSMA-ligand PET.
RECIP	Response Evaluation Criteria in PSMA Imaging	Specifically designed for PSMA PET, these criteria evaluate treatment effectiveness in metastatic castration-resistant prostate cancer by focusing on new lesions and overall PSMA tumor volume changes.

**Table 3:** Summary of clinical trials assessing 177Lu-PSMA-617 therapy in prostate cancer.

Clinical Trial Identifier/Authors	Study Objective	Phase	Status
VISION (NCT03511664)	177Lu-PSMA-617 and Soc versus Soc alone in mCRPC	3	Complete
UpfrontPSMA (NCT04343885)	177Lu-PSMA-617 followed by docetaxel versus docetaxel	2	Active, not recruiting
LuTectomy (NCT04430192)	177Lu-PSMA-617 before radical prostatectomy and pelvic lymph node dissection	1/2	Active, not recruiting
LuPSMA (ACTRN12615000912583)	177Lu-PSMA-617 in mCRPC	2	Complete
TheraP (NCT03392428)	177Lu-PSMA617 versus cabazitaxel in mCRPC	2	Complete
PSMAfore (NCT04689828)	177Lu-PSMA-617 vs. change in ART in taxane-naïve mCRPC	3	Active, not recruiting
